# Effects of Dietary Protein Levels on Growth, Digestive Enzyme Activity, Antioxidant Capacity, and Gene Expression Related to Muscle Growth and Protein Synthesis of Juvenile Greasyback Shrimp (*Metapenaeus ensis*)

**DOI:** 10.3390/ani13243886

**Published:** 2023-12-18

**Authors:** Fei Xiao, Jiawei Wang, Huaming Liu, Minjia Zhuang, Xiaobo Wen, Huihong Zhao, Kun Wu

**Affiliations:** 1 College of Marine Sciences, South China Agricultural University, Guangzhou 510642, China; xiaofei@stu.scau.edu.cn (F.X.); 18143604051@163.com (J.W.); 15118767339@163.com (H.L.); minjiazhuang@outlook.com (M.Z.); wenxbo@scau.edu.cn (X.W.); 2 Fisheries Research Institute of South China Agricultural University, Nansha, Guangzhou 510642, China

**Keywords:** *Metapenaeus ensis*, dietary protein, growth performance, digestive enzyme, antioxidant, muscle growth, protein synthesis

## Abstract

**Simple Summary:**

Greasyback shrimp (*Metapenaeus ensis*) is a highly sought-after shrimp species in the market. However, there is a lack of studies investigating the optimal dietary protein level for greasyback shrimp, which hampers the development of shrimp farming for this species. Therefore, this study aimed to explore the effects of dietary protein levels on various aspects of growth, antioxidant capacity, digestive enzyme activity, and gene expression related to muscle growth and protein synthesis in juvenile greasyback shrimp. The ultimate goal was to determine the optimal protein requirement for these shrimp. The findings revealed that the appropriate dietary protein level for juvenile greasyback shrimp is 38.59%, as determined by the broken-line regression analysis of the specific growth rate (SGR). These results serve as a foundation for the development of formulated diets tailored for greasyback shrimp.

**Abstract:**

An 8-week feeding trial was conducted to assess the effects on growth, antioxidant capacity, digestive enzyme activity, and gene expression related to muscle growth and protein synthesis of juvenile greasyback shrimp (*Metapenaeus ensis*) using five experimental diets containing 29.37%, 34.30%, 39.11%, 44.05%, and 49.32% of protein. The results demonstrated that juvenile greasyback shrimp consuming 39.11%, 44.05%, and 49.32% dietary protein had a significantly higher final body weight (FBW), weight gain (WG), feed conversion ratio (FCR), and specific growth rate (SGR) than other groups (*p* < 0.05). The protein efficiency ratio (PER) showed a significantly quadratic pattern with increasing dietary protein levels *(p* < 0.05). The highest trypsin and pepsin activities were observed in the group with a protein level of 44.05% (*p* < 0.05). Relatively higher superoxide dismutase (SOD) activity was found in groups with protein levels of 39.11% (*p* < 0.05). Alkaline phosphatase (AKP) and catalase (CAT) activity showed a significantly linear increasing pattern with increasing protein intake up to 44.05%, and then decreased gradually (*p* < 0.05). Compared to the dietary 29.37% protein level, the expression levels of myogenic regulatory factors (*mef2α*, *mlc*, and *myf5*) and mTOR pathway (*mtor*, *s6k*, *akt*, and *pi3k*)-related genes were significantly up-regulated in muscle with 39.11%, 44.05%, and 49.32% dietary protein levels (*p* < 0.05). The AAR pathway (*gcn2*, *eif2α*, and *atf4*)-related gene expression levels were significantly lower in muscles with 39.11%, 44.05%, and 49.32% protein levels than in other groups (*p* < 0.05). Based on the broken-line regression analysis of SGR, the estimated appropriate dietary protein requirement for juvenile greasyback shrimp is 38.59%.

## 1. Introduction

Shrimp is the most valuable commodity in the seafood industry, accounting for 16.4% of the total trade value [1]. However, China’s shrimp production heavily relies on a single species, Penaeus vannamei, which poses a risk of vulnerability to diseases and environmental changes [2]. Greasyback shrimp (*Metapenaeus ensis*) is a promising alternative for shrimp farming in China. This species can thrive in both saltwater and freshwater conditions, and it has a high protein content and a good taste [3]. However, the lack of basic nutritional research on greasyback shrimp hinders the development of specific feed and large-scale cultivation.

Protein plays a crucial role in the growth and production of aquatic animals, as it is a major component of their bodies [4]. Given the high cost of fishmeal, protein accounts for a significant portion of feed expenses. Additionally, proteins have essential functions in cell construction, renewal, repair, energy supply, and the production of biologically active substances [5]. Therefore, maintaining appropriate dietary protein levels is not only important for optimal shrimp growth but also for reducing feed costs [6]. In addition, excessive protein intake can lead to increased catabolism, elevated nitrogen waste, and potential health and growth issues [7,8]. Thus, finding the right balance of dietary protein is crucial for achieving optimal growth and economic benefits. Studies have shown that dietary protein levels can influence enzyme activity in shrimp [9,10]. For example, Xia et al. [7] observed an increase in digestive enzyme activity in *Litopenaeus vannamei*, while Talukdar et al. [11] found reduced enzyme activity with higher protein levels in the diet. Since the activity of digestive enzymes affects nutrient accessibility and utilization, studying these enzymes is essential for understanding digestion mechanisms and determining nutritional requirements [12,13].

Protein regulates muscle growth, which affects body composition and growth performance [14]. The process of muscle growth heavily relies on myostatin (MSTN) and myogenic regulatory factors (MRFs) such as MyoG, Myf5, and MyoD [15]. Studies have shown that a high-protein diet increases MSTN synthesis in the muscles of mammals and fish [16,17]. Furthermore, optimal dietary protein levels led to the growth of myofibers in the sub-adult grass carp (*Ctenopharyngodon idella*) [18]. These findings demonstrated that dietary protein likely affects myofiber development through the regulation of MSTNs and MRFs, which has not yet been studied in shrimp. In addition, the amino acid response (AAR) and target of rapamycin (mTOR) pathways are considered to play a role in modulating growth and protein metabolism in some aquatic animals [19,20,21]. Thus, exploring the effects of dietary protein on the AAR and TOR pathways contributes to the elucidation of potential mechanisms regulating the growth performance of juvenile greasyback shrimp.

Therefore, this study investigated the effects of dietary protein on growth, antioxidant capacity, digestive enzyme activity, and gene expression related to muscle growth and protein synthesis of juvenile greasyback shrimp, and the objective was to determine the optimal protein requirement for juvenile greasyback shrimp. These findings enhanced our understanding of the nutritional requirements and metabolic mechanisms of juvenile greasyback shrimp and provided a reference for the formulation of juvenile greasyback shrimp feeds.

## 2. Materials and Methods

### 2.1. Experimental Diets

Based on the protein levels of diets determined by previous studies [22,23,24], this study formulated diets with different graded inclusion levels of crude protein (actual protein levels: 29.37, 34.30, 39.11, 44.05, and 49.32 g/100 g), as shown in Table 1. The diets were labeled P29, P34, P39, P44, and P49, respectively. The main protein sources used were fishmeal, gelatin, casein, and soybean meal, while soybean oil and fish oil served as lipid sources. All ingredients were ground and weighed according to the feed formulation ratios, mixed thoroughly, and moistened with oil and water. The mixture was extruded into pellets 1.60 mm in diameter using a twin-screw extruder (TSE65, Beijing Yanggong Machinery Technology Developm/ent Co., Ltd., Beijing, China). The amino acid composition of all experimental diets is presented in Table 2.

### 2.2. Experimental Procedure

The experiments were undertaken at Nansha Agricultural Group, a commercial outdoor pond (50 m × 60 m × 2.5 m) in Guangzhou, Guangdong, China. The pond water had a low salinity of 5‰. Juvenile greasyback shrimp (*Metapenaeus ensis*) were obtained from a local hatchery and acclimated to the pond conditions for 14 days in net cases (1 m × 1 m × 1.5 m; 3 mm mesh) before the feeding trial. During the adaptation stage, the shrimp were fed commercial feeds.

The experiment was conducted in 15 net cages (1 m × 1 m × 1.5 m; 3 mm mesh) suspended in the outdoor pond, with a 1 m gap between each cage. Healthy juvenile shrimps with a mean original weight of 1.92 ± 0.02 g were randomly assigned to the cages, with 100 shrimps per cage and three cages per treatment. Experimental diets were given to the shrimp twice a day at 6:00 and 19:00. The feeding rate ranged from 3% to 6% of the total shrimp weight and was adjusted based on weather conditions, shrimp growth, and feeding behavior. The experiment lasted for 56 days. The water parameters of pH, ammonia-N, dissolved oxygen, and nitrite were measured regularly and maintained within the suitable ranges for juvenile greasyback shrimp, with dissolved pH ranging from 7.63 to 8.29, nitrite ranging from 0.02 to 0.13 mg/L, ammonia-N ranging from 0.1 to 0.4 mg/L, and oxygen ranging from 4.52 to 9.12 mg/L.

### 2.3. Sample Collection

After 8 weeks of culture, the shrimp were fasted for 24 h and then sampled. The shrimp were anesthetized on ice for 10 min, weighed, and the total weight of each cage of shrimp was recorded to calculate the survival rate (SR), feed conversion ratio (FCR), weight gain (WG), and specific growth rate (SGR). The individual weight and body length of each shrimp were also measured. Muscle and hepatopancreas samples were collected from 15 shrimps randomly selected from each cage for muscle composition and digestive enzyme activities.

### 2.4. Analysis Method of Nutritional Components

According to previous studies, the approximate composition of diets and muscle tissues was analyzed [25]. The samples were oven-dried at 105 °C until a constant weight was reached to determine their moisture content. The contents of crude lipid and crude protein in muscle and diets were measured by the Soxhlet system and the Kjeldahl method (Xianjian Instruments, Shanghai, China), respectively. The composition of free amino acids was contained by an automatic amino acid analyzer in shrimp muscle and diets (Hitachi L-8900, Hitachi, Tokyo, Japan).

### 2.5. Biochemical Indexes and Digestive Enzyme Analysis

The hepatopancreas samples were homogenized with a PBS solution at a 1:9 weight ratio on ice. Then, the sample suspension was homogenized in an ice bath with a homogenizer and centrifuged (800× *g*, 10 min) at low temperature, and the supernatant was aspirated to test enzyme activity and antioxidant index analyses. Biochemical indexes and digestive enzyme analysis were conducted following the commercial kit instructions (Nanjing Jiancheng Bioengineering Co., Ltd., Nanjing, China). Before conducting formal experiments, pre-experiments were performed to ensure appropriate enzyme kinetics.

### 2.6. Quantitative Real-Time Polymerase Chain Reaction

Using the RNA isolater Total RNA Extraction Reagent (Vazyme Biotech Co., Ltd., Nanjing, China) to extract the total RNA from muscle and intestinal samples. Electrophoretic separation of 1.5% denaturing agarose gel and a spectrophotometer (GE Pharmacia, Fremont, CA, USA) were used to evaluate the quality and concentration of the extracted RNA. Subsequently, the RNA was reverse transcribed into cDNA using the reverse transcription kit (Takara Corporation, Kusatsu, Japan). As the manufacturer instructed, the reaction was carried out in 2 μL of cDNA diluted fivefold, 4 μL (2 μM) of each forward and reverse primer, and 10 μL of TOROGreen^®^ qPCR Master Mix (Toroivd Technology Co., Ltd., Shanghai, China). The amplification program consisted of an initial step at 95 °C for 60 s, followed by 40 cycles of 95 °C for 10 s and 60 °C for 30 s. A final melt curve analysis was conducted from 60 °C to 95 °C, with increments of 0.5 °C for 0.05 s. Using *ef1α* as the housekeeping gene, the 2^−ΔΔCT^ algorithm was used to calculate the relative mRNA expression of each target gene compared to the relative mRNA expression of the P29 dietary group as the control group. Table 3 lists the specific primers.

### 2.7. Statistical Analysis

The values were calculated with the following formulae:

Weight gain (WG, %) = (final body weight − initial body weight) × 100/initial body weight.

Feed conversion ratio (FCR) = dry feed fed (g)/wet Weight gain (g).

Specific growth rate (SGR, %/day) = 100 × (Ln (final body weight) − Ln (initial body weight))/days.

Hepatosomatic index (HSI, %) = 100 × hepatopancreatic weight(g)/body weight (g).

Condition factor (CF, g/cm^3^) = 100 × body weight (g)/body length (cm^3^).

Protein efficiency ratio (PER) = wet weight gain (g)/dry protein fed (g).

Survival rate (SR, %) = 100 × (final number of shrimp/initial number of shrimp).

Statistical analyses were performed with SPSS 27.0 software. The results were displayed as means ± S.E.M (standard error of means). Prior to statistical analysis, all data were evaluated for normality using the Kolmogorov–Smirnov test. Barlett’s test was performed to test the homogeneity of variances. Then, data were subjected to one-way ANOVA followed by Turkey’s multiple-range tests. *p* < 0.05 was regarded as significant. Moreover, in order to determine whether the significant effects were linear and/or quadratic, a follow-up trend analysis using orthogonal polynomial contrasts was performed. Additionally, a broken-line regression analysis was used to determine the optimal dietary protein level. The analysis was conducted with R 3.0.1 (R Development Core Team 2013) based on the relevant R codes reported by Lee et al. [26].

## 3. Results

### 3.1. Growth Performance and Feed Utilization

**Figure 1:**
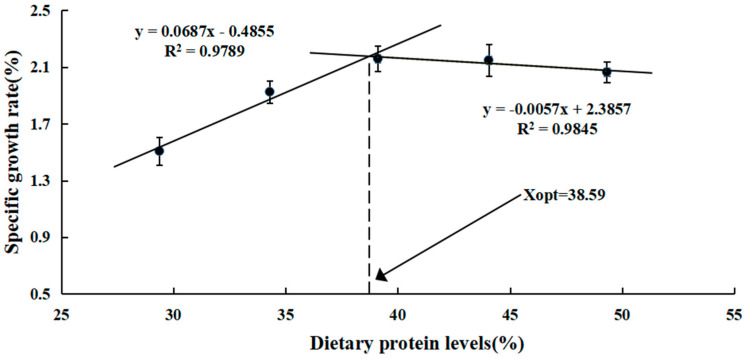
Relationship between specific growth rate (SGR) and dietary protein levels based on broken−line regression analysis, where Xopt represents the optimal dietary protein level for achieving maximum SGR. Each point represents the mean of three replicates.

Greasyback shrimp fed different levels of protein were analyzed for growth performance and feed utilization in Table 4. As dietary protein levels increased, the final body weight (FBW), specific growth rate (SGR), and weight gain (WG) of juvenile greasyback shrimp showed significant linear and quadratic increasing trends (*p* < 0.05), but no significant differences were noticed among the P39, P44, and P49 groups (*p* > 0.05). The feed conversion ratio (FCR) significantly declined as dietary protein levels increased and then remained stable (*p* < 0.05). In the P29 group, the condition factor (CF) was significantly lower compared to the other groups (*p* < 0.05). The protein efficiency ratio (PER) showed a significantly quadratic pattern with increasing dietary protein levels, peaking at the P39 group (*p* < 0.05). However, the survival rate (SR) and the hepatosomatic index (HSI) were not strikingly altered by changes in dietary protein intake (*p* > 0.05). Based on the broken-line regression analysis of the specific growth rate (SGR) (Figure 1), the estimated optimal dietary protein requirement for juvenile greasyback shrimp is 38.59%.

### 3.2. Digestive Enzyme Activity in Hepatopancreas

The effects of dietary protein levels on the hepatopancreatic digestive enzyme activity of juvenile greasyback shrimp are reported in Table 5. In the P39 and P44 groups, trypsin activity in the hepatopancreas was significantly higher than in the other groups (*p* < 0.05). Pepsin activity increased in a significantly linear and quadratic pattern with dietary protein levels from 29.37% to 44.05% (*p* < 0.05). α-amylase activity decreased significantly linearly with increasing protein levels (*p* < 0.05), peaking at the P29 group with significance (*p* < 0.05). There were no significant effects of dietary protein on lipase activity (*p* > 0.05).

### 3.3. Hepatopancreas Antioxidant Parameters

The effects of dietary protein levels on the hepatopancreas antioxidant parameters are presented in Table 6. Alkaline phosphatase (AKP) and catalase (CAT) activity in the hepatopancreas exhibited a significant linear increasing pattern with increasing protein intake up to 44.05%, and then gradually decreased (*p* < 0.05). Malondialdehyde (MDA) content in the hepatopancreas was significantly reduced when the dietary protein level ranged from 39.11% to 49.32% (*p* < 0.05). The activity of superoxide dismutase (SOD) in the hepatopancreas shrimp showed a significantly quadratic increasing pattern with dietary protein levels from 29.37% to 39.11%, and then progressively decreased (*p* < 0.05). 

### 3.4. Muscle Proximate Composition and Amino Acid Composition Analysis

The nutrient composition of the muscle is presented in Table 7. As the dietary protein level increased, muscle crude protein exhibited a significant increase followed by a decrease (*p* < 0.05), reaching a maximum value of 84.32% in the P39 group. However, there was no significant effect of dietary protein on muscle moisture, ash, and crude lipid (*p* > 0.05).

The amino acid composition of the muscle is shown in Table 8. No significant difference was found between the groups in terms of the amount of lysine, cysteine, and histidine (*p* > 0.05). The content of lysine was the highest among the essential amino acids. The contents of threonine, valine, isoleucineand, arginine, serine, glutamic acid, alanine, tyrosine, and methionine showed a significant linear and quadratic relationship with dietary protein levels (*p* < 0.05). The contents of total essential amino acids (TEAA) and total nonessential amino acids (TNEAA) in the muscle of juvenile greasyback shrimp were significantly lower in the P29 group compared to the other groups (*p* < 0.05). 

### 3.5. Gene Expression of mTOR and AAR Pathways in Muscle

**Figure 2:**
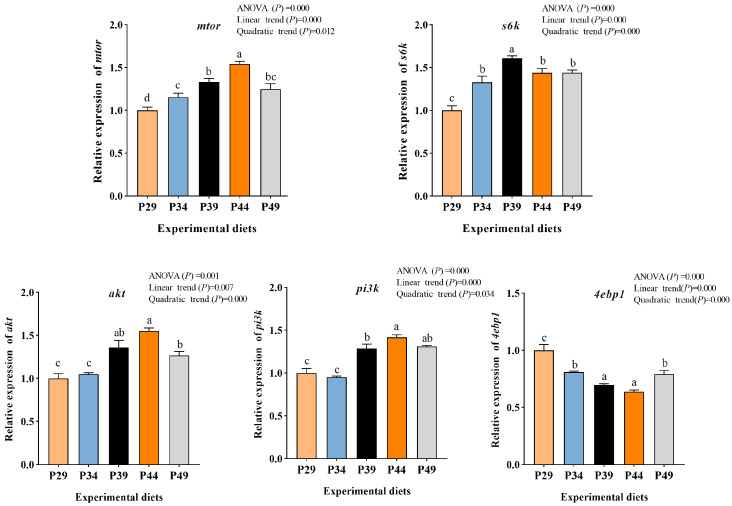
Relative mRNA expression of mTOR pathway in muscle of juvenile greasyback shrimp (*Metapenaeus ensis*) fed different diets. Data are presented as means ± S.E.M (*n* = 3). Different lowercase letters above the bars represent significant differences (*p* < 0.05).

The results indicated that dietary protein levels significantly impacted the expression of critical genes in the mTOR signaling pathway in the muscle of juvenile greasyback shrimp (Figure 2) (*p* < 0.05). Among these genes, *mtor*, *s6k*, *akt*, and *pi3k* displayed a pattern of initially increasing and then declining as the dietary protein level increased (*p* < 0.05). The gene expression of *mtor*, *akt*, and *pi3k* reached its peak in the P44 group, while the gene expression of *s6k* reached its highest level in the P39 group. Meanwhile, the gene expression of *4ebp1* showed a significant linear and quadratic relationship with dietary protein levels (*p* < 0.05), and it was significantly lower in the P39 and P44 groups compared to other groups (*p* < 0.05).

**Figure 3:**
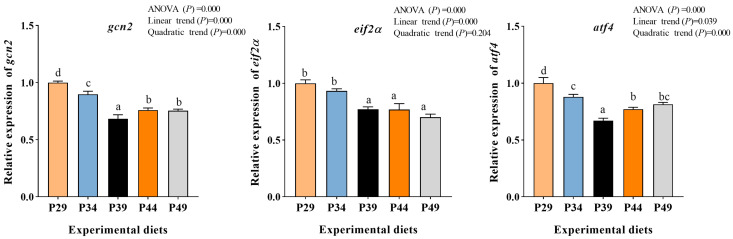
Relative mRNA expression of AAR pathway in muscle of juvenile greasyback shrimp (*Metapenaeus ensis*) fed different diets. Data are presented as means ± S.E.M (*n* = 3). Different lowercase letters above the bars represent significant differences (*p* < 0.05).

The gene expression of *eif2α* displayed a significant linear decreasing pattern with increasing protein levels (*p* < 0.05). The highest expression levels of *eif2α* were observed in the groups fed 29.37% and 34.30% protein. The gene expression of *gcn2* and *atf4* in the AAR pathway decreased sharply with increasing dietary protein levels up to 39.11% and then increased slowly (*p* < 0.05). The group fed 39.11% protein had the lowest expression levels of *gcn2* and *atf4* (Figure 3).

### 3.6. Gene Expression of Myogenic Regulatory Factors in Muscle

**Figure 4:**
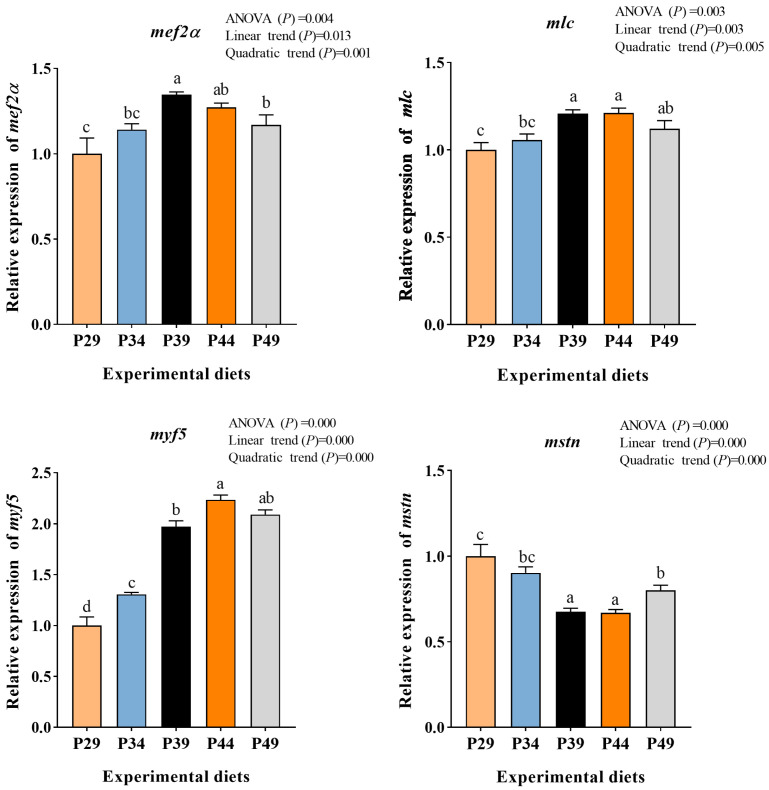
Relative mRNA expression of myogenic regulatory factors in muscle of juvenile greasyback shrimp (*Metapenaeus ensis*) fed different diets. Data are presented as means ± S.E.M (*n* = 3). Different lowercase letters above the bars represent significant differences (*p* < 0.05).

The gene expression of *mef2α*, *mlc*, and *myf5* showed an initial rise followed by a decline as the dietary protein level increased. Additionally, as compared with P29 and P34 groups, these three genes were significantly more expressed in P39 and P44 groups (*p* < 0.05). The gene expression of *mstn* showed a significant linear and quadratic relationship with dietary protein levels (*p* < 0.05). Compared to other groups, the gene expression of *mstn* was strikingly lower in P39 and P44 groups (*p* < 0.05) (Figure 4).

## 4. Discussion

In this study, the growth performance of juvenile greasyback shrimp (*Metapenaeus ensis*) was significantly influenced by different levels of dietary protein. As dietary protein intake increased, the final body weight (FBW), specific growth rate (SGR), and weight gain (WG) showed significant linear and quadratic increasing trends. However, no further improvement in growth was observed with higher dietary protein levels. Analogous results have been recorded in other crustaceans, such as juvenile whiteleg shrimp (*Litopenaeus vannamei*) [22], Chinese mitten crab (*Eriocheir sinensis*) [27], and mud crab (*Scylla paramamosain*) [28]. Under the given conditions, the optimal protein requirement for juvenile greasyback shrimp was approximately 38.59%, as determined by the broken-line regression analysis of SGR. Previous studies have established the dietary protein requirements for different shrimp species, such as 30–35% for Indian white shrimp (*Penaeus indicus*) [23], 33.4% for juvenile whiteleg shrimp (*Litopenaeus vannamei*) [22], and 37% for oriental river prawn (*Macrobrachium nipponense*) [24]. The variation in results may be influenced by shrimp species, growth stage, nutritive quality of the feed protein source, and the experimental environment. In the present study, the protein efficiency ratio (PER) showed a significantly quadratic pattern with increasing dietary protein levels. This finding is consistent with other studies [27,29,30]. The PER decreased in the low-protein group (P29 group) of juvenile greasyback shrimp, possibly due to lower protease activity compared to the other groups. This lower activity resulted in weaker digestion of dietary proteins in the low-protein group and thus a lower PER. Moreover, PER was reduced in the high-protein group (P49 group) of juvenile greasyback shrimp, likely due to excessive protein consumption for energy [27]. In this study, a low dietary protein level produced a higher FCR, which may be due to the fact that the level of nutrients consumed was not sufficient to promote growth, as has been observed in some other studies [31,32].

Several prior studies on other crustacean species, such as pacific white shrimp (*Litopenaeus vannamei*), mud crab (*S. serrata*), and swimming crab (*P. trituberculatus*)*,* have shown that dietary protein had a positive influence on body protein content [33,34,35]. An analogous result was also found in the present study where the highest muscle protein content was obtained for the dietary protein level of 39.11%. In addition, it was observed that the crude lipid content in mud crab (*S. serrata*) increased with increasing dietary protein. However, this study showed that muscle crude lipid and ash content were not significantly affected by different dietary protein levels. An analogous result was obtained in the Pacific white shrimp *Litopenaeus vannamei* [33]. Furthermore, with increasing dietary protein levels, muscle total essential amino acid (TEAA) content was maximized in the P39 group. The result indicated that the excess essential amino acids in shrimp fed with high-protein diets are directed to catabolism and energy production [36]. In contrast, low-protein diets lead to amino acid deficiency, resulting in insufficient protein synthesis in shrimp muscle, which in turn leads to low muscle protein content [34]. In this study, muscle nonessential amino acids (NEAA) content increased significantly with increasing dietary protein levels. The protein requirements of animals are determined by their need for essential amino acids, as well as their non-specific need for amino acid groups and nitrogen for the synthesis of nonessential amino acids and other metabolic needs of the animal [37]. TEAA was significantly lower in the P29 group compared to the other groups, indicating that the lack of protein in the diet can be detrimental to the quality of tissue. In addition, it was observed that the crude lipid content in mud crab *S. serrata* increased with increasing dietary protein. However, this study showed that muscle crude lipid and ash content were not significantly affected by different dietary protein levels. An analogous result was obtained in the Pacific white shrimp *Litopenaeus vannamei* [33]. 

The growth performance of animals is closely related to their ability to utilize nutrients, and digestive enzyme activities play a crucial role in nutrient absorption, digestion, and utilization [38,39]. In this study, a negative correlation was found between dietary protein levels and α-amylase activity. The high α-amylase activity observed in juvenile greasyback shrimp indicated efficient starch digestion, which could be attributed to the high level of dietary carbohydrates in low-protein diets [40]. Similar increases in α-amylase activity were also observed in Eriocheir sinensis when fed higher levels of carbohydrates [27]. The activities of trypsin and pepsin showed significant linear and quadratic relationships with dietary protein levels, which is consistent with the findings of Xia et al. [7]. These results suggest that optimal protein levels can increase protease activity and improve the digestibility of juvenile greasyback shrimp. It is evident that animals can adapt their digestive enzymes according to their diet, thereby enhancing their growth rate. Moreover, when fed reduced levels of protein in this study, protease activity was also reduced, likely due to the lower quantity of dietary protein available as a substrate for protease activity, which aligns with the findings of Giri et al. [40].

The antioxidant enzymes and phosphatases are critical for non-specific immunity and have been extensively assayed to assess the health of crustaceans [41,42]. Phosphatases are primarily found in the hepatopancreas and are involved in various metabolic processes, including calcium uptake, chitin secretion, and calcium phosphate deposition [28]. In this study, alkaline phosphatase (AKP) activity exhibited a significant linear increase with increasing protein intake, peaking at 44.05% (*p* < 0.05). Additionally, superoxide dismutase (SOD) and catalase (CAT) are important antioxidant enzymes to protect organisms from oxidative stress [43]. The hepatopancreatic SOD and CAT activities of juvenile greasyback shrimp were significantly increased with dietary protein levels of 39.11% and 44.05%, respectively. Moreover, the supply of moderate protein (39.11% to 49.32%) significantly reduced the concentration of malondialdehyde (MDA) in the hepatopancreas of juvenile greasyback shrimp. The higher level of MDA was found to have higher cytotoxicity and accelerated the damage of cells and tissues [44]. These results indicate that proper protein levels in the diet can improve the health status of juvenile greasyback shrimp.

The growth of aquatic animals, particularly in muscle tissues, is primarily driven by protein deposition through protein synthesis [45]. It is recognized that mammalian target of rapamycin (mTOR) and amino acid response (AAR) pathways are key pathways for sensing nutrients, regulating protein synthesis, and downstream metabolism in cells [46]. The activated mTOR stimulates translation initiation by activating *s6k* and inhibiting *4ebp1*. In this study, a low-protein diet led to decreased gene expression of *mtor* and *s6k* and increased gene expression of *4ebp1* in muscle. This may be attributed to insufficient substrates such as amino acids needed for protein synthesis. Amino acids play a crucial role in activating the mTOR pathway [47]. Interestingly, the muscle on a low-protein diet exhibited significantly lower TEAA and TNEAA contents compared to the other groups, which may contribute to the downregulation of the mTOR pathway. The mTOR pathway is known to be positively regulated by the PI3K/AKT pathway [48]. In the present study, the optimum dietary protein upregulated the gene expression of *akt* and *pi3k* in muscle, indicating that the activated mTOR pathway on optimal dietary protein may be partially ascribed to the upregulation of gene expression of *akt* and *pi3k*. This finding aligns with the research conducted by Ma et al. [49]. The AAR pathway regulates translation initiation, which is a limiting step in protein synthesis [50]. Former findings in teleost have indicated that imbalances of amino acids could lead to the activation of the AAR pathway [51]. In this study, the low-protein diet upregulated the expression of AAR pathway-related genes, *gcn2*, *atf4*, and *eif2α*, which may be attributed to the amino acid deficiency caused by the low-protein diet. Research has indicated that an appropriate amino acid diet can promote growth performance by inhibiting the AAR pathway [51]. Therefore, optimal dietary protein can activate the mTOR pathway and inhibit the AAR pathway, promoting muscle protein synthesis and enhancing the growth performance of juvenile greasyback shrimp.

The degree of muscle development is influenced by nutritional status [52]. Previous studies have demonstrated that myofiber development is regulated by various muscle regulatory factors, which directly control myofibrillogenesis [53]. At the molecular level, several myogenic regulatory factors (MRFs) regulate myofiber growth. In this study, appropriate dietary protein intake significantly upregulated the muscle gene expression of *mef2α*, *mlc*, and *myf5*, indicating that appropriate dietary protein may promote myofiber growth by regulating the gene expression of MRFs in shrimp muscle. Mstn, on the other hand, negatively regulates myofiber growth by inhibiting proliferation and myogenic differentiation in C2C12 myoblasts [54]. Interestingly, the optimal dietary protein level in this study reduced the gene expression of mstn, suggesting that dietary protein could enhance muscle growth by decreasing the gene expression levels of mstn. However, it should be noted that a high-protein diet increased the gene expression of mstn, which is consistent with the findings in male Wistar rats [16]. This suggests that when dietary protein exceeds the animal’s requirements, mstn gene expression may be upregulated to prevent excessive muscle hypertrophy. Overall, the results indicate that an optimal dietary protein level may enhance muscle growth in shrimp by regulating MRFs and MSTN gene expression. However, further research is needed to develop a more detailed model of protein regulation of gene expression related to muscle growth in shrimp.

## 5. Conclusions

The current findings demonstrate that an appropriate dietary protein level has positive effects on growth, muscle composition, antioxidant capacity, digestive enzyme activities, muscle development, and protein synthesis of juvenile greasyback shrimp. Furthermore, the muscle protein content increased as the dietary protein levels increased. This increase can be attributed to the upregulation of genes related to myogenic regulatory factors (*mef2α*, *mlc*, and *myf5*) and the mTOR pathway (*mtor*, *s6k*, *akt*, and *pi3k*), as well as the downregulation of genes related to the AAR pathway (*gcn2*, *eif2α*, and *atf4*). Based on the broken-line regression analysis of the specific growth rate (SGR), the optimum dietary protein requirement for juvenile greasyback shrimp is approximately 38.59%. These findings provide a solid foundation for the development of formulated diets for this shrimp species.

## Figures and Tables

**Table 1:** Composition and nutrient content of experimental diet (dry matter, g/100 g).

Ingredients	Dietary Protein Levels				
	P29	P34	P39	P44	P49
Fish meal (67.7% protein)	27.00	27.00	27.00	27.00	27.00
Soybean meal (45.5% protein)	20.00	20.00	20.00	20.00	20.00
Casein (87.3% protein)	1.20	5.70	10.20	14.70	19.20
Gelatin (91.5% protein)	0.30	1.43	2.55	3.68	4.80
Squid meal	50.00	50.00	50.00	50.00	50.00
α-starch	23.00	17.00	12.00	7.00	2.00
Oil ^a^	4.60	4.60	4.60	4.60	4.60
Lecithin powder	2.00	2.00	2.00	2.00	2.00
Vitamin C phosphate	0.20	0.20	0.20	0.20	0.20
Vitamin premix ^b^	1.00	1.00	1.00	1.00	1.00
Mineral Premix ^c^	1.00	1.00	1.00	1.00	1.00
Choline chloride	0.50	0.50	0.50	0.50	0.50
Ca(H_2_PO_3_)_2_	1.00	1.00	1.00	1.00	1.00
CMC-Na	1.50	1.50	1.50	1.50	1.50
Celluose	11.70	12.08	11.45	10.83	10.20
Proximate composition (g/100 g dry matter)					
Crude Protein	29.37	34.30	39.11	44.05	49.32
Crude Lipid	7.08	7.12	7.15	7.19	7.22
Ash	11.30	11.56	11.58	11.85	11.96

Notes: Fish meal, Soybean meal, Casein, and Gelatin were provided by Guangzhou Kemu Biotechnology Co., Ltd., Guangzhou, China. ^a^ Oil consisted of a mixture of soybean oil and fish oil with a ratio of 1:1. ^b^ Per kilogram of vitamin premix: vitamin A, 120.0 mg; vitamin B1, 3.5 g; vitamin B2, 1.5 g; vitamin B6, 3.0 g; vitamin B12, 10.0 mg; vitamin D3, 112.5 mg; vitamin E, 1.5 g; vitamin K3, 1.0 g; niacinamide, 0.75 g; inositol, 3.6 g; calcium-pantothenate, 1.7 g; biotin, 150 mg; folic acid, 2.5 g. ^c^ Per kilogram of mineral premix: MgSO_4_·7H_2_O, 5 g; FeSO_4_·7H_2_O, 20 g; NaSeO_3_, 20 mg; KI, 80 mg; CuSO_4_·5H_2_O, 900 mg; ZnSO_4_·7H_2_O, 2 g; CoCl_2_·6H_2_O, 100 mg; MnSO_4_·H_2_O 600 mg.

**Table 2:** Amino acid compositions of the experimental diets (dry matter, g/kg).

Index	Experimental Diets				
	P29	P34	P39	P44	P49
Essential amino acids					
Threonine	12.28	13.18	14.13	14.98	15.53
Valine	14.70	16.14	17.48	18.06	19.19
Methionine	6.23	6.90	7.61	7.91	8.09
Isoleucine	12.75	14.43	15.11	15.91	16.67
Leucine	21.90	23.20	25.87	27.11	28.01
Phenylalanine	16.58	17.70	18.51	19.23	19.66
Lysine	16.20	18.34	20.47	21.56	22.69
Histidine	7.10	7.79	8.78	9.26	9.93
Arginine	16.13	17.56	18.89	19.98	20.81
Total essential amino acids	123.85	135.22	146.84	153.99	160.56
Nonessential amino acids					
Aspartic acid	24.28	26.89	28.86	30.81	32.15
Serine	13.38	14.22	14.70	15.78	15.89
Glutamic acid	48.02	53.61	55.74	57.70	58.34
Glycine	14.03	15.45	16.16	17.59	18.44
Alanine	14.05	15.98	16.46	18.03	18.91
Cysteine	4.15	3.86	4.03	5.01	5.27
Tyrosine	8.70	9.82	10.16	11.02	11.64
Proline	14.93	15.87	16.07	16.34	16.77
Total nonessential amino acids	141.52	155.69	162.16	172.25	177.39
Total amino acids	265.37	290.91	308.99	326.24	337.95
TEAA/TAA	0.47	0.46	0.48	0.47	0.48

Notes: Tryptophan was not determined due to the acid hydrolysis. Abbreviations: TEAA, total essential amino acids; TNEAA, total nonessential amino acids; TAA, total amino acids; TEAA/TAA, ratio of total essential amino acids to total amino acids.

**Table 3:** Primer sequences of target genes in real-time PCR analysis.

Target Gene	Primer Sequence Forward (5′-3′)	Primer Sequence Reverse (5′-3′)	Accession Number
*4ebp1*	AGAGTGCGGTCGTGAATG	ACGCTGTGGTGTTGTGGT	XM_037925357.1
*akt*	CAGATGGCTGGGAAATAC	TAAGGAAACGCTGACGAA	XM_043035795.1
*pi3k*	GCACGGAAAATGATGTCT	CCTCTGTGTTTGGGTTGA	XM_037935655.1
*s6k*	GCCCTTCTCCCCTCGTCTT	GGTTGCTCGTGTCCATCTG	XM_027368997.1
*mtor*	GTCTGCTGAAAACGCTAC	CGGATTCTACAATGATGG	XM_037943752.1
*atf4*	GCCCACGGCATTATCCTTC	CCTTCCCACCAACTTCACA	XM_043001354.1
*eif2α*	TCGCCCTATCGCCTCAAGG	CGACAATACCCGCTCCGCA	XM_037943017.1
*gcn2*	TCCCGACCACAAAGAACAT	AGAACAAGGCAGCAACGAT	XM_037934051.1
*mef2α*	TGCCGCCCTCTGTACCTCT	CACCACCACCACACCACGA	XM_047633265.1
*mlc*	AGCCAACCGCTACACCCCT	ACAGTCTCGCCCTCCTCCA	XM_037925429.1
*mstn*	GGAGATAACTGGGGAGGAG	GGTGGAACTGAGGAAGAAG	MG437236.1
*myf5*	CTTACACTGTGAAGCAATA	GCACGAGAAGTAGAAACGA	XM_037918242.1
*ef1α*	ATGTGTGTGGAGACCTTCC	CACCTGCTTCCTTCTTGTT	MG775229.1

Notes: Abbreviations: *4e-bp1*, 4E binding protein; *akt*, protein kinase B; *pi3k*, phosphatidylinositol 4,5-bisphosphate 3-kinase catalytic subunit delta isoform; *s6k*, ribosomal S6 protein kinase; *mtor*, mammalian target of rapamycin; *atf4*, activating transcription factor 4; *eif2α*, eukaryotic translation initiation factor 2; *gcn2*, general control nonderepressible 2; *mef2*, myocyte enhancer factor 2; *mlc*, myosin light chain; *mstn*, myostatin; *myf5*, myogenic factor 5; *ef1α*, eukaryotic initiation factor 1.

**Table 4:** Growth parameters and feed utilization of juvenile greasyback shrimp (*Metapenaeus ensis*) fed experimental diets.

Index	Experimental Diets					ANOVA (*p*)	Linear Trend (*p*)	Quadratic Trend (*p*)
	P29	P34	P39	P44	P49			
Initial body weight (g)	1.92 ± 0.19	1.91 ± 0.11	1.92 ± 0.13	1.93 ± 0.15	1.91 ± 0.18	0.962		
Final body weight (g)	4.47 ± 0.14 ^a^	5.62 ± 0.11 ^b^	6.44 ± 0.12 ^c^	6.39 ± 0.08 ^c^	6.10 ± 0.11 ^c^	0.000	0.002	0.000
Weight gain (%)	132.81 ± 7.41 ^a^	194.07 ± 5.75 ^b^	235.24 ± 6.35 ^c^	233.02 ± 5.40 ^c^	217.88 ± 5.56 ^c^	0.000	0.000	0.003
Feed conversion ratio	1.89 ± 0.02 ^c^	1.45 ± 0.07 ^b^	1.19 ± 0.05 ^a^	1.20 ± 0.04 ^a^	1.20 ± 0.03 ^a^	0.000	0.000	0.001
Specific growth rate (%/d)	1.51 ± 0.06 ^a^	1.93 ± 0.03 ^b^	2.16 ± 0.03 ^c^	2.15 ± 0.03 ^c^	2.07 ± 0.03 ^c^	0.000	0.000	0.000
Hepatosomatic index (%)	2.53 ± 0.23	3.49 ± 0.51	3.46 ± 0.04	2.89 ± 0.10	2.91 ± 0.03	0.099	0.850	0.187
Condition factor (g/cm^3^)	0.81 ± 0.05 ^a^	0.90 ± 0.02 ^b^	0.97 ± 0.01 ^b^	0.89 ± 0.01 ^b^	0.89 ± 0.01 ^b^	0.012	0.064	0.004
Protein efficiency ratio (%)	1.67 ± 0.09 ^a^	2.01 ± 0.06 ^bc^	2.16 ± 0.06 ^c^	1.89 ± 0.04 ^b^	1.71 ± 0.02 ^a^	0.000	0.635	0.000
Survival rate (%)	80.67 ± 0.02	83.00 ± 0.02	81.00 ± 0.01	79.00 ± 0.01	81.00 ± 0.06	0.644	0.573	0.957

Notes: Data are presented as means ± S.E.M (standard error of means). Values not sharing a common superscript in the same row differ significantly (*p* < 0.05).

**Table 5:** Digestive enzyme activity in hepatopancreas of juvenile greasyback shrimp (*Metapenaeus ensis*) fed experimental diets.

Index	Experimental Diets					ANOVA (*p*)	Linear Trend (*p*)	Quadratic Trend (*p*)
	P29	P34	P39	P44	P49			
Trypsin (U/mg prot)	3817.41 ± 70.24 ^a^	4612.33 ± 40.42 ^b^	5210.15 ± 80.17 ^c^	5333.10 ± 137.71 ^c^	4780.63 ± 102.80 ^b^	0.000	0.003	0.002
Pepsin (U/mg prot)	5.71 ± 0.6 ^a^	6.43 ± 0.34 ^ab^	8.48 ± 0.35 ^b^	10.85 ± 1.00 ^c^	10.62 ± 0.23 ^c^	0.013	0.000	0.005
α-Amylase (U/mg prot)	0.80 ± 0.01 ^d^	0.63 ± 0.02 ^c^	0.50 ± 0.02 ^b^	0.39 ± 0.01 ^ab^	0.35 ± 0.01 ^a^	0.000	0.001	0.124
Lipase (U/mg prot)	0.71 ± 0.45	0.88 ± 0.16	0.77 ± 0.08	0.84 ± 0.06	0.81 ± 0.31	0.101	0.750	0.152

Notes: Data are presented as means ± S.E.M (standard error of means). Values not sharing a common superscript in the same row differ significantly (*p* < 0.05).

**Table 6:** Antioxidant parameters in the hepatopancreas of juvenile greasyback shrimp (*Metapenaeus ensis*) fed experimental diets.

Index	Experimental Diets					ANOVA (*p*)	Linear Trend (*p*)	Quadratic Trend (*p*)
	P29	P34	P39	P44	P49			
Alkaline phosphatase (U/mg prot)	32.04 ± 3.59 ^a^	43.41 ± 3.39 ^a^	63.97 ± 4.45 ^bc^	71.57 ± 2.46 ^c^	58.13 ± 4.96 ^b^	0.001	0.017	0.154
Catalase (U/mg prot)	12.36 ± 0.54 ^a^	15.38 ± 0.79 ^b^	17.91 ± 0.52 ^c^	19.67 ± 0.51 ^d^	18.87 ± 0.62 ^cd^	0.000	0.005	0.327
Malondialdehyde (nmol/mg prot)	5.94 ± 0.51 ^c^	4.56 ± 0.28 ^b^	3.21 ± 0.34 ^a^	3.43 ± 0.55 ^a^	3.51 ± 0.31 ^a^	0.021	0.064	0.000
Superoxide dismutase (U/mg prot)	35.05 ± 3.65 ^a^	47.58 ± 6.34 ^b^	61.85 ± 3.98 ^d^	57.30 ± 4.81 ^c^	52.59 ± 4.09 ^bc^	0.000	0.167	0.001

Notes: Data are presented as means ± S.E.M (standard error of means). Values not sharing a common superscript in the same row differ significantly (*p* < 0.05).

**Table 7:** Muscle proximate compositions of juvenile greasyback shrimp (*Metapenaeus ensis*) fed experimental diets (% wet weight).

Index	Experimental Diets					ANOVA (*p*)	Linear Trend (*p*)	Quadratic Trend (*p*)
	P29	P34	P39	P44	P49			
Moisture (%)	77.23 ± 0.10	77.85 ± 0.34	77.52 ± 0.47	77.61 ± 0.25	76.65 ± 0.13	0.392	0.182	0.388
Crude protein (%)	17.08 ± 0.18 ^a^	17.13 ± 0.13 ^b^	17.66 ± 0.14 ^c^	17.53 ± 0.19 ^c^	17.17 ± 0.22 ^b^	0.000	0.062	0.000
Crude lipid (%)	2.10 ± 0.46	2.03 ± 0.53	2.15 ± 0.51	2.28 ± 0.58	2.30 ± 0.38	0.112	0.296	0.763
Ash (%)	1.31 ± 0.62	1.35 ± 0.78	1.29 ± 0.69	1.21 ± 0.77	1.38 ± 0.82	0.455	0.096	0.274

Notes: Data are presented as means ± S.E.M (standard error of means). Values not sharing a common superscript in the same row differ significantly (*p* < 0.05).

**Table 8:** Muscle amino acid compositions of juvenile greasyback shrimp (*Metapenaeus ensis*) fed experimental diets (g/kg of wet weight).

Index	Experimental Diets					ANOVA (*p*)	Linear Trend (*p*)	Quadratic Trend (*p*)
	P29	P34	P39	P44	P49			
Essential amino acids								
Threonine	6.20 ± 0.22 ^a^	6.34 ± 0.19 ^b^	6.47 ± 0.18 ^c^	6.48 ± 0.33 ^c^	6.31 ± 0.27 ^b^	0.001	0.043	0.007
Valine	8.28 ± 0.45 ^a^	8.53 ± 0.55 ^b^	8.67 ± 0.65 ^b^	8.58 ± 0.43 ^b^	8.40 ± 0.35 ^ab^	0.018	0.024	0.009
Methionine	2.52 ± 0.21 ^a^	2.56 ± 0.27 ^b^	2.62 ± 0.15 ^c^	2.61 ± 0.24 ^c^	2.64 ± 0.21 ^c^	0.011	0.000	0.035
Isoleucine	7.39 ± 0.35 ^a^	7.64 ± 0.33 ^b^	7.78 ± 0.42 ^b^	7.67 ± 0.51 ^b^	7.58 ± 0.31 ^b^	0.000	0.047	0.001
Leucine	10.76 ± 0.35 ^a^	11.20 ± 0.51 ^b^	11.40 ± 0.35 ^c^	11.21 ± 0.51 ^b^	10.88 ± 0.54 ^a^	0.001	0.096	0.000
Phenylalanine	7.52 ± 0.31 ^a^	7.72 ± 0.38 ^b^	7.90 ± 0.39 ^c^	7.81 ± 0.41 ^b^	7.61 ± 0.41 ^ab^	0.007	0.005	0.052
Lysine	12.20 ± 0.60	12.62 ± 0.72	12.90 ± 0.43	12.67 ± 0.59	12.38 ± 0.58	0.128	0.270	0.096
Histidine	5.53 ± 0.38	5.57 ± 0.21	5.71 ± 0.28	5.56 ± 0.27	5.50 ± 0.39	0.084	0.319	0.195
Arginine	11.02 ± 0.85 ^ab^	11.36 ± 0.84 ^c^	11.50 ± 0.78 ^c^	11.26 ± 0.91 ^b^	10.93 ± 0.74 ^a^	0.011	0.048	0.014
Total essential amino acids	70.74 ± 4.83 ^a^	74.85 ± 5.54 ^b^	75.92 ± 5.72 ^c^	75.07 ± 4.87 ^c^	72.35 ± 5.36 ^b^	0.000	0.003	0.000
Nonessential amino acids								
Aspartic acid	17.17 ± 2.02 ^a^	17.71 ± 2.12 ^b^	17.96 ± 1.57 ^b^	17.83 ± 2.31 ^b^	17.36 ± 1.08 ^a^	0.001	0.325	0.000
Serine	4.71 ± 0.28 ^a^	4.96 ± 0.15 ^bc^	4.90 ± 0.21 ^b^	5.04 ± 0.25 ^c^	4.85 ± 0.31 ^a^	0.002	0.004	0.017
Glutamic acid	26.17 ± 2.52 ^a^	27.25 ± 3.31 ^bc^	27.39 ± 2.14 ^c^	27.11 ± 3.52 ^b^	26.38 ± 3.28 ^a^	0.013	0.037	0.003
Glycine	15.20 ± 0.35 ^b^	15.30 ± 0.45 ^b^	15.15 ± 0.67 ^ab^	15.09 ± 0.48 ^a^	14.90 ± 0.52 ^a^	0.005	0.008	0.083
Alanine	11.02 ± 0.88 ^a^	11.54 ± 0.75 ^c^	11.64 ± 0.62 ^c^	11.41 ± 0.83 ^b^	11.50 ± 0.75 ^c^	0.000	0.035	0.012
Cysteine	3.43 ± 1.72	3.30 ± 1.32	3.68 ± 1.67	3.35 ± 1.59	3.48 ± 1.77	0.128	0.158	0.253
Tyrosine	3.21 ± 0.52 ^a^	3.41 ± 0.41 ^c^	3.42 ± 0.57 ^c^	3.35 ± 0.64 ^b^	3.26 ± 0.53 ^a^	0.000	0.011	0.001
Proline	11.12 ± 0.43 ^a^	11.64 ± 0.57 ^c^	12.18 ± 0.64 ^d^	11.52 ± 0.55 ^b^	11.48 ± 0.67 ^b^	0.000	0.217	0.002
Total nonessential amino acids	92.76 ± 3.32 ^a^	95.47 ± 3.61 ^bc^	97.19 ± 2.95 ^c^	94.65 ± 4.05 ^b^	93.21 ± 3.55 ^b^	0.000	0.043	0.000
Total amino acids	162.49 ± 6.51 ^a^	167.25 ± 5.84 ^b^	172.46 ± 6.67 ^c^	166.52 ± 5.98 ^b^	164.67 ± 6.38 ^ab^	0.001	0.024	0.000

Notes: Data are presented as means ± S.E.M (standard error of means). Values not sharing a common superscript in the same row differ significantly (*p* < 0.05).

## Data Availability

All data are contained within the article.
